# Sort of a nice distance: a qualitative study of the experiences of therapists working with internet-based treatment of problematic substance use

**DOI:** 10.1186/s13722-019-0173-1

**Published:** 2019-11-27

**Authors:** Veronica Ekström, Magnus Johansson

**Affiliations:** 1 The Department of Social Sciences, Ersta Sköndal Bräcke University College, Stockholm, Sweden; 2 Department of Public Health Sciences, Karolinska Institutet, Stockholm, Sweden; 3 Stockholm Center for Dependency Disorders, Stockholm Health Care Services, Stockholm County Council, Stockholm, Sweden

**Keywords:** Alcohol use disorder, Substance use disorder, Web-based intervention, eHealth, Therapeutic alliance, Qualitative research, Focus groups

## Abstract

**Background:**

Internet interventions have been developed and tested for several psychiatric and somatic conditions. Few people with substance use disorders receive treatment and many drug users say that they would prefer getting help from online tools. Internet interventions are effective for reducing alcohol and cannabis use. The aim of the current study is to understand differences between internet-based and face-to-face treatment of problematic substance use. The concept of alliance will be used as a theoretical frame for understanding differences between internet-based treatment and face-to-face treatment, as perceived by therapists.

**Method:**

The study has a qualitative design and is based on 3 focus group interviews with 12 therapists working with internet-based treatment for alcohol or cannabis use problems within five different programs.

**Results:**

The analysis revealed five themes in the differences between internet-based and face-to-face treatment: communication, anonymity, time, presence and focus. Treatment online in written and asynchronous form creates something qualitatively different from regular face-to-face meetings between patients and therapists. The written form changes the concept of time in treatment, that is, how time can be used and how it affects the therapist’s presence. The asynchronous (i.e. time delayed) form of communication and the lack of facial expressions and body language require special skills.

**Conclusions:**

There are important differences between internet-based treatment and face-to-face treatment. Different aspects of the alliance seem to be important in internet-based treatment compared to face-to-face.

## Background

Internet interventions, online treatment, computer-assisted therapy, web-based treatment, e-health… there is a plethora of terms for describing interventions which take place online instead of in the more traditional setting, where a counselor or a therapist meets a patient face to face. Wide-spread access to computers, internet and smartphones has facilitated both development and implementation of interventions. Internet delivered cognitive behavioral therapy (ICBT) has been developed and tested for several psychiatric and somatic conditions [1]. Within the field of substance use disorders, reviews have shown that internet-based interventions are effective at reducing alcohol [2, 3] and cannabis use [4]. Different categories can be identified within the field of internet interventions: self-administered therapy or pure self-help, predominately self-help, minimal contact therapy and predominantly therapist administered therapy [5]. Therapist supported internet interventions seem to work better than self-help interventions [6, 7]. In a study that investigated the experiences of users of an internet-based self-help program aimed at reducing alcohol consumption, the perceived privacy of the internet was important in searching for help and to avoid stigma and embarrassment [8]. During the development of the same intervention, users expressed appreciation of the self-help exercises and the non-judgmental tone of texts [9].

A systematic review on expectations and experiences regarding e-health treatment with focus on women [10] provide an overview of perceived disadvantages and advantages of internet-based treatment outside of the substance use field. Internet-based treatment require more self-discipline and motivation from the users, in order to avoid skipping parts of the treatment. The delay in time, and absence of non-verbal information, can disrupt the communication between the user and the therapist and make it feel less empathic. Writing text messages might make it more difficult for users to explain complex situations and feelings and lead to misunderstandings. At least some of the patients in most previous studies say they miss face-to-face contact or that they would have liked closer contact with their therapist. The users enjoy the flexibility of internet-based treatment with easy access without traveling, and availability at any time; it can be fitted into their every-day life. The personal responsibility that comes with this flexibility is appreciated and can give users a sense of autonomy and a feeling of being empowered. The relationship with an online therapist is viewed as important and sometimes experienced as just as close as in face-to-face treatment [10].

In a meta-synthesis of user experiences, the level of contact and level of independence are described as important factors in computerized therapy [11]. Some patients are unable or unwilling to accept ICBT without interpersonal contact because they feel left alone, while others appreciate the enhanced anonymity and flexibility of the treatment and feel secure, with the majority of patients being ambivalent [12]. In interviews with both clients and counselors, Dunn found a unique use of time within asynchronous counseling and that “time to think” was a critical theme differentiating online counseling from traditional [13]. Psychology students describe that the asynchronous communication in ICBT is beneficial for learning [14].

The collaborative and conscious aspects of the relationship between a therapist and a patient is referred to as alliance [15]. The panteoretical concept of alliance was developed by Luborwsky and Bordin [16]. According to Bordin, alliance is based on the cooperation and shared view between patient and therapist and is composed of three components: the bond (mutual trust and acceptance), the agreement about the goals of therapy, and the agreement about the tasks for therapy [17]. Alliance has been shown to have an impact on outcome in psychological interventions, regardless of psychotherapeutic orientation [15, 18]. Alliance can be as strong and have similar impact on outcomes in internet-based interventions, even though the format is different [19–21]. The anonymity and the way of communicating as an online therapist, affect the development of the alliance [22]. In a recent study on experience of ICBT, therapists expressed that alliance may be achieved faster and more easily in face-to-face therapy [23]. According to a review on alliance in internet interventions, future studies should try to identify unique characteristics of alliance in different treatment formats [20].

Estimations show that only about 7% of people with substance use disorders receive treatment [24]. Some possible reasons for people not seeking substance use treatment are shame and stigma, wanting to deal with the problems on their own or poor access to treatment [25, 26]. Internet interventions might help address these problems. A recent Global Drug Survey show that people in in English speaking countries, without severe problems, now prefer getting help from online tools [27]. Few studies have investigated potential adverse effects [28] and relatively little is still known regarding mediators of change [2] in internet interventions aimed at reducing the use of alcohol and other substances. Studies using qualitative methods can be helpful in investigating potential benefits and disadvantages of internet-based treatments. Exploring the experience of therapists as well as users can help understand the mechanism of change. Studying internet-based treatment also gives an opportunity to better understand how the internet might change the practice of therapy and counseling [29]. To our knowledge, no study to date has investigated how therapists experience working with Internet-based treatment aimed at reducing substance use.

The aim of the current study is to understand differences between internet-based and face-to-face settings in the treatment of problematic use of alcohol, problematic use of cannabis or family members of people with problematic alcohol use. The concept of alliance [17] will be used as a theoretical frame for understanding differences in internet-based treatment and face-to-face treatment, as perceived by therapists.

The following research questions will be addressed in the article:

1. What differences between face-to-face treatment and internet-based treatment for problematic substance use do therapists describe?
2. What do these differences mean for alliance in treatment?

## Material

The study has a qualitative design, using a thematic analysis [30]. People working with internet-based treatment for alcohol and cannabis (called therapists from now on) within five different programs were asked if they could participate in a group interview. To our knowledge, these five programs were the only existing internet-based treatment programs for alcohol and cannabis in Sweden at the time. Common for the five programs, apart from being internet-based, is that they are all manual-based programs within a CBT-tradition, with added elements of motivational interviewing (MI). The programs address people who want to quit or reduce their consumption of either alcohol (eChange [31], ePlus and Alkoholhjälpen) cannabis (Cannabishjälpen) or have a close family member with alcohol use-disorder (eCRAFT). The therapists who worked with the alcohol and family programs also have interacted with users in a well-established online discussion-forum that is offered publicly independent of program participation.

All therapists working in the five programs were invited to participate in the study. 12 of 14 agreed to participate (see Table 1). Three group interviews were conducted by author 1 during 2017. Participants were mixed so that they would be interviewed together with therapists from different programs and not only with their colleagues. A conference room in the treatment clinic responsible for all treatment programs in the study, was used. However, this was not the physical work place for all therapists. A semi-structured interview guide including questions regarding differences between face-to-face treatment and internet-based treatment was used (Additional file 1: Interview guide). Examples of questions are *How* does *technology affect your work? What do you think is different with internet-based treatment? Do you as therapists need to do something different or be different?* Follow-up questions were formulated continuously during the interviews. All three interviews started with a description of the aim of the study and an explanation of the group interview and research ethics. All participants introduced themselves with name, occupation and professional background. The interviewer emphasized that consensus among participants was not a goal and they were encouraged to speak freely. The interviews lasted for approximately 90 min and were recorded and transcribed verbatim. Transcripts were then sent to the participants for comments and/or corrections.

**Table 1:** Brief description of the 12 interviewed therapists

IP	Education	Gender	Program	Current work^a^
11	Psychology	Woman	Alcohol	Face-to-face
12	Psychology, Ph.D.	Man	Alcohol	Internet
13	Social work	Woman	Cannabis	Face-to-face
14	Behavioral science	Woman	Alcohol	Internet and face-to-face
21	Psychology, Ph.D.	Man	Alcohol	Research
22	Psychology	Man	Alcohol	Internet
23	Social work	Woman	Cannabis	Face-to-face
31	Psychology	Man	Alcohol, family	Face-to-face
32	Social work	Woman	Alcohol	Internet
33	Psychology, Ph.D.	Woman	Alcohol	Research
34	Psychology	Woman	Family	Face-to-face
35	Psychology	Woman	Alcohol, family	Face-to-face

^a^The kind of therapeutic work that the informant has worked with after being a therapist in respective study

- Interview session 1: IP11, IP12, IP13 and IP14.
- Interview session 2: IP21, IP22 and IP23.
- Interview session 3: IP31, IP32, IP33, IP34 and IP35.

The transcribed interviews were read by both authors and preliminary themes were identified. The interviews were then coded by author 1 in Open Code, a software for qualitative coding, following Braun and Clarke’s guidelines for thematic analysis [30]. The codes used were developed during the coding and from what the interviewees talk about. The coding process has moved back and forth between the three interviews. Examples of codes that were developed are *text*, *anonymity, physical attributes*, *availability* and *relation.* The next step of the analysis was to read the material code by code. Again, the analysis has alternated between the full interview and reading code by code. In this process, we introduced the concept of alliance in our analysis and used it to deepen our understanding of the therapists’ experiences of working with internet-based treatment. Five major themes were finally constructed: Communication, Anonymity, Time, Presence and Focus. To facilitate the assessment of this study’s credibility, a significant number of quotes are presented in order to increase transparency in the analysis. The quotes chosen have been so because they serve as the most vivid or illustrative examples in capturing the essence of a certain topic [30]. We have followed the COREQ checklist for reporting on qualitative research (Additional file 2: COREQ checklist).

We use the term “therapist” for all the respondents in our study. They are a mixture of mostly psychologists and trained social workers. They could be called therapists, counsellors, advisors, social workers, etc. depending on education and setting. They are all experienced in treating alcohol and drug related problems.

Most of the time we use internet-based treatment as a concept for different kinds of treatment or programs that take place online. “Traditional” treatment is called face-to-face treatment in the article. We are aware of the fact that interned-based treatment could be given face-to-face online, through e.g. Skype or video chats. However, this is not the kind of treatment our interviewees have experiences from.

The interviewees in this study have different ways to describe the people they work with. Some of them talk about their clients. Others talk about users or participants. They can also be described as patients. We have chosen to use the term “patient” in this article even though users might in fact be the better term. In most programs covered in this study, they are not clients or patients in a formal sense. They have signed up to participate in a study and have used an internet-based program, perhaps even anonymously. However, the term “user” can be confusing in the context of alcohol- and drug treatment, since it is a common term for the person who uses alcohol or drugs.

## Results

We have constructed five major themes in our analysis, and we will present our analysis by using these themes as headings: communication, anonymity, time, presence and focus.

### Communication

The therapists in this study have all worked in settings where written methods are used. The patient logs on to the website and receives written instructions on how to complete a certain module in the program. The patient then answers written questions by writing responses or reflections to a theme. The therapist reads the patient’s responses and responds in writing with comments or additional questions. If there is contact apart from this, it is also in writing, through e-mails or discussion-forums. Treatment is therefore internet-based, but also very much text-based. It is the written language that constitutes both the therapist’s and the patient’s tool, not spoken language as in treatment that take place face-to-face.

The use of written language entails specific challenges. As some of the therapists mention, it puts demands on the patient’s writing skills. This suits some of the patients, but not all. Patients with poor language skills or who only write short comments are harder to work with, which the therapist in the following quote gives an example of:

*If you ask “Did you feel like this?” And then get response… in writing, you never get that so you must guess, and you almost never get an answer that is a confirmation on if you were right or wrong. It’s also very difficult with those who write very briefly, that you must, well you can’t guess, you have to remain within the limits of reason. If you get very little text to work with you can’t guess that much (IP35).*

Communication in writing differs from verbal communication, as the quote above illustrates. To ask a quick question to get confirmation is something the therapist might do in face-to-face treatment, but it is more difficult in internet-based treatment. Brief written statements can also be hard to interpret. To be able to give meaningful feedback, the therapist needs adequate written material to reflect on. Naturally, as the therapist in the quote below points out, the opposite also occurs. There are patients that express themselves better in writing, compared to face-to-face conversations:

*Not everyone finds words so easy either. It’s not everyone that are verbal in that way and think it’s easy to express themselves. To respond immediately to a question that is thrown out (IP22).*

Conducting alcohol- or drug treatment online and in text-based form, deprives the therapists of important tools they have access to in face-to-face treatment, namely body language, facial expressions and other visible characteristics within the patient. The quote below is an example of how this is described in the interviews:

*Our patients are anonymous, but they have filled in quite a lot of scoring forms, so we’ve got that information. Which can actually be more than you have when you meet someone in real life, someone who comes to your office. But I….at the same time…you don’t have this feeling in the room or that you see the person and the immediate reaction to what you say (IP23).*

The following quote is from another therapist who also describes the lack of facial expressions:

*I think the most important difference is that you don’t get an immediate response to what you say. You don’t have a face to look at. You don’t know if you have tuned in at the right level, if you have been too convoluted, too childish, too academic or so, until you have been working a while and developed a joint lingo with the patient (IP34).*

The therapist describes an uncertainty that occurs when they do not have access to the subtle information they have in have in face-to-face treatment. It is hard for them to know how the things they write are then perceived by the patient. The therapist in the quote below talks about this in a similar way:

*Here you read what the person writes and chooses to add and then I respond to that. It´s about using language in a completely different way compared to when you speak because then I can use gestures, I can use tone. Here I must deliver it so that this person understands that I’m interested, I want to know what you think, what it is that you’re experiencing as troublesome or what questions you have, without pressuring for information (IP13).*

The therapists emphasize the importance of finding a way to be personal even though they never meet the patient and only communicate online. It is described as something different from what they do as a therapist in a face-to-face conversation:

*There are different ways to do that in a conversation and perhaps some other ways to do it in writing. (…). It comes down to finding that…way to show that you see that it’s a person. So that they don’t think we’re just sitting here and answering like a robot. (…) You find something you send back, something that indicates some sort of processing (IP21).*

As with patients, the written format suits some therapists more than others. They must also be comfortable with writing instead of talking. Among the interviewed therapists there are individuals who are more comfortable and pleased with the written format than others, due to personal skills and preferences. One of the therapists says face-to-face sessions in general are more demanding:

*To see how someone looks when you say something also puts different demands on you. If someone gets really sad or very anxious, you can’t keep on with the manual. (…) I get much more tired from having face-to-face sessions compared to working on the internet. It demands more of me. More presence (IP23).*

### Anonymity

As described earlier, some of the programs that the interviewees have worked in are anonymous. The patient creates an alias and the therapists do not know their real identity. Anonymity is seen as a good method to reach people in need and perhaps also motivate them to seek more treatment if they need it. The following quote is an example of this:

*For many people, it’s a steppingstone to seek face-to-face treatment and have the courage to tell it as it is. That is the big advantage, I think. When you (the patient, our remark) write and you know that this person (the therapist, our remark) doesn’t know who I am, I’m free to write what I’m most ashamed of, the things I think is most troublesome and you can’t get to me (IP13).*

Anonymity in cannabis programs might be of special relevance, due to the illegal status of cannabis use in Sweden. One therapist, who has worked within a cannabis program, is surprised over how much information patients reveal when they get the opportunity to be anonymous:

*I’ve sometimes been surprised by some patients in the cannabis program, that they have told me about very tricky things…that I sort of didn’t expect. But perhaps they haven’t found any other place to do so. Maybe they test me a bit, what will happen if I say this or that they sort of get the courage to open up a bit when it comes to certain aspects. I think it has to do with the fact that they are anonymous and that I can’t do anything (IP23).*

The therapists describe anonymity as an important feature of internet-based interventions that increase self-disclosure and disinhibition from the patients. The therapists themselves are not anonymous, but in the anonymous programs they mostly introduce themselves by first name only. When asked why, one therapist answers:

*In the program, we have only been our first names. Actually, it´s a bit odd when you think about it. There is no reason, I mean we could just as well be our full names (IP34).*

Another therapist responds and explains that for her, to introduce herself with first name only, is a way to enable the patient to remain anonymous. If she would introduce herself with her full name, perhaps the patient would feel a pressure to do so as well. Remaining informal by not using your full name can be used as a strategy in internet-based treatment.

### Time

The written format also has consequences for time, according to the analysis in this study. The therapists talk about time in different ways as a component of what constitutes internet-based treatment. In face-to-face treatment, a therapist might meet the patient for e.g. 45 min every or every second week. Contact in between sessions rarely takes place. Apart from writing memory notes in the patient’s journal, the therapist does not work actively with the patient in between sessions. Furthermore, communication in face-to-face treatment is immediate in the sense that when a question is raised, it is most of the time expected that an answer is given in response. This is how verbal communication normally takes place.

In internet-based treatment, technology forms treatment, and specifically, timing in treatment. What can be described as rhythm or pace in treatment seems to be more diverse in internet-based forms than in traditional settings. As described by IP34 in a quote above, the lack of immediate response to what they have written is one of the most important differences compared to face-to-face treatment. On the other hand, the therapists do not have to give an immediate response either:

*You have much more possibility for reflection. It´s not as fast and not the same…perhaps not the same demand to deliver in the moment. You can think for a while, go and talk to someone, ask for input if you need (IP23).*

The possibility to reflect and even talk to colleagues before writing a response to your patient is mentioned by several of the therapists as one of the advantages with internet-based treatment. Some of the therapists also describe how the written form in some cases makes the treatment “flat” or that they experience a sort of “muteness”. It can take several days before they get a response from the patient on something they have written or a question they have asked. This delay in time is what can make the treatment a bit “flat”. In the following quote, one of the therapists talk about differences between face-to-face treatment and internet-based treatment:

*I can only feel…I’m sorry, the muteness in the program. When I sit with someone live who has done an assignment and I feel it was a bit flat or not done so thorough, you can continue to work with it when you meet. I feel like this delay that occurs when you’re not chatting live but are sending a message, sometimes you must leave things that are not really done thoroughly. You could perhaps have analyzed it more (IP11).*

A side-effect of the written form is that treatment is documented, and this also add to certain aspects of time. Both therapists and patients can go back to previous assignments or conversations whenever they want:

*As a patient, you can always go back to something written. It´s preserved in a different way than a conversation that is more of a being something fresh (…) A lot of people write that they will go back and read both previous tasks and our conversations. It kind of continuous, I think (IP22).*

When the written material is available, it is possible to reflect upon it on numerous occasions, which might benefit treatment, as the therapist in the quote above describes. But the preserved written material can also have disadvantages if the therapist perhaps has misunderstood something or have written something that the patient misunderstands. This is exemplified in the quote below:

*The written text is both an advantage and a disadvantage. The text continues to exist, and the patient can log on 14 times a day and read what I wrote. It’s amazing if it was spot on and if it wasn’t that spot on at all…it´s not so good. The advantage, but also the disadvantage, with face-to-face treatment is that of 45 minutes, they only remember maybe three minutes (IP11).*

### Presence

The combination of CBT-inspired treatment and the written, internet-based form seems to have an impact on therapist presence in treatment. Some of the therapists use distance to describe something negative that occurs in the relation with the patient in internet-based treatment. Others describe it as a positive effect. The following conversation between two of the therapists is an example of this:

*It can be quite comfortable for me as a therapist because it doesn’t catch on. I have, of course, still a great responsibility, but for me, it’s easier to go home for the day and not think about the people I work with. The patients. (…) There is a relief in that. I like it (IP32).*

*Sort of a nice distance (IP31).*

*Yes. I don’t know if I would call it distance, but perhaps that’s what it is (IP32).*

Some of the therapists say they take up less space in internet-based treatment, compared to face-to-face treatment. This is probably an effect of both the written aspects and the time aspects that is evident in internet-based treatment. The therapist in the quote below describes difficulties in being certain that the patient receives necessary information and at the same time, the importance of not drowning the patient in writing:

*You can kill any patient by text just because the therapist wants to deliver all he or she has. It’s really difficult to not try to add everything you have just to sort of be sure that the patient has got what he or she needs. Yes, it’s difficult (IP34).*

Even though the therapists take up less space in treatment, i.e. are less present, they are more present in terms of availability. The written format and the internet-based form mean more frequent contact compared to a face-to-face patient, whom the therapist sees once every week or every second week. How often the therapist communicates with patients in internet treatment differs. It is a natural consequence of the idea that the patient should do the program in his or her own pace. However, there is an agreement that the therapist answers in a couple of days. Beyond giving response to assignments, the therapists also describe that they communicate with patients if necessary:

*Actually, they get much more contact. Sometimes you chat, if you’re working at the computer, then I answer if there is a question. A perfect face-to-face patient comes once every second week and there is nothing in between. You can have a lot of contact with an internet-based patient (IP22).*

As the therapist in the quote below says, communication online enables more contact between patient and therapist, but there is a risk with constant availability:

*On the one hand, we’re more available and can answer messages even if I’ve written that I will answer on Tuesdays. You can answer on other days as well. And then you’re much more available than with face-to-face patients. Still, you can start to think like, well I didn’t answer that simple question at 10 p.m. No, because I don’t work. There is a sort of limitlessness in how available you can be (IP14).*

The program, with its written material and exercises, and the discussion forum, where patients can have an ongoing discussion, also provide a sort of presence of treatment, even though the therapist is not present. In the quote below one of the therapists exemplifies this by using the metaphor of moving in with the patient:

*One of the great benefits is that something can, sort of, move in with the patients with internet. We are there, even if you don’t answer on a Friday night, the program is there, and they can look at what you’ve written earlier and see your comments. (…) I can miss that in face-to-face treatment (IP11).*

That the therapist takes up less space in treatment also contributes to greater autonomy for the patient, according to some of the therapists. Time and technology are also important factors that increases autonomy in internet-based treatment, according to some of the therapists. The following quote is an example of how it is expressed in the interviews:

*The benefit is that it’s so much on the user’s terms, I think. Both when it comes to time and place and what you want to tell and not (IP23).*

Patients can choose when to use the program, and use it in the setting of their choice. They can easily stop treatment by just not logging on to the website anymore. The therapists say there are higher drop-out rates among their patients online compared to patients who participate in face-to-face treatment. The patient seems to be in charge of their treatment to a greater extent and, as the therapist in the quote below says, the internet-based format combined with the specific programs emphasize patients’ own responsibilities:

*It’s a higher degree of own responsibility that is evident in the contact, I think. You sort of…it’s more self-help (IP22).*

Earlier in the interview the same therapist (IP22) says that as a therapist, you must be okay with the high degree of autonomy within internet-based treatment, since it is an aspect that affects your work.

### Focus

So far, increased autonomy for the patient, taking up less space and being more available﻿ as a therapist have been described as important aspects of presence in internet-based treatment. How focus is affected is a related theme. According to the therapists in this study, internet-based treatment also affects focus in treatment. One therapist explains that it’s “easier to stick to the content and not talk about other things” (IP22). The therapist in the quote below discusses small talk and how the physical body might disturb focus in face-to-face treatment:

*I think it’s easier to be more focused on what the patient seeks help for. When you meet someone face-to-face it’s always small talk about weather and it can be different things. You notice it on appearances that have changed. It’s so much with the physical actually, and you get the body out of the way, so to speak. And the weather…so it’s, what do you want? What do you need? (IP33).*

Even though the therapists seem to agree on that internet affects focus in treatment, there are differences in how they interpret this. For some, it is described as a benefit that makes their work easier and more pleasant and an important aspect since it increases autonomy for the patients. Other seem to have a more critical approach. For example, the therapist in the quote below, describes differences between face-to-face and internet-based treatment and according to the therapist, there is a risk that you lose depth in analysis:

*The program gets very stream-lined on internet. With face-to-face patients, even if the content is the same, I experience a greater variation in their own personal stories about alcohol and their life-story. I’ve thought about some of them, I’ve had some very special life stories in face-to-face treatment. If they had been randomized to internet-based treatment, they would have been one of many in this mass who had set a goal to cut down to two glasses on Friday and two glasses on Saturday and we had identified risk factors and made a plan…they would have sort of passed through. In face-to-face treatment, we’ve been able to explore much more on what they had gone through and how they had ended up here and what was relevant (IP11).*

As the quote above illustrates, a consequence that might be negative is that the strict focus on the program and the written material prevents both the patient and the therapist to work with aspects that are also important for the patient’s treatment or well-being. The therapist in the quote below describes this in a similar way. There is a risk of missing important aspects that are in fact connected to the patient’s problematic use of alcohol or cannabis:

*I’m thinking about how often I wanted to, because the questions came about a lot of stuff that didn’t have to do directly to it, but my task was that no matter what, no matter if there were questions, back to the program! I mean, the program can be useful. I was very guided by it. But me as a person, with my competence, I could have done much more. I wanted to do more (…). There were many open doors I wanted to go in to with the patient and think about, because of course it’s connected to why they are here (IP32).*

The therapist above is expressing a sort of frustration over the restraints of the program and the strict focus it gives to the treatment. Some of the therapists also describe a concern over that severe social problems, i.e. violence against adults or children, are not identified within online forms of treatment and potentially go unnoticed.

## Discussion

In this study, we have analyzed the perspectives of therapists working with substance use disorders both online and face-to-face. We identified five categories where differences between these two ways of delivering treatment exist; communication, anonymity, time, presence and focus. Communication in writing differs from verbal communication. There is a lack of immediate response but increased time for reflection in internet-based treatment. The perceived anonymity is important and can increase self-disclosure and disinhibition from the patients. Therapists experience less presence and take up less space in internet-based treatment but are more available. Internet-based programs increase focus on treatment content rather than other problems of the patient. In this final part we will discuss our findings. We start with differences between internet-based treatment and face-to-face treatment, followed by a discussion on what these differences might mean for alliance.

Many of the differences between internet-based and face-to-face treatment described by the therapists in this study are in line with the experience of internet-based treatment as described by patients in previous research [10, 11]. The same differences might be regarded as both positive and negative. The description of how the written form and time delay affect the communication is in line with a previous research stating that the lack of visual cues and face expressions can be a problem in internet-based treatment [32] and that the potential for misunderstandings are bigger [10]. But the therapists in this study also describe positive aspects of the way of communicating similar to Dunn [13] who found a unique use of ‘time’ within the asynchronous counselling and that “time to think” was a critical theme differentiating online counselling from traditional. The possibility to reflect or talk to colleagues before they write a response to a patient is mentioned by therapists as one of the advantages with internet-based treatment. This is in line with benefits from training in guided ICBT with asynchronous communication reported by students [14]. In line with users’ experiences from an internet-based program for alcohol [8], the therapists in this study highlight the importance of anonymity. The possibility to be anonymous can reduce social barriers to self-disclosure, which might be extra important for people who have experienced stigma or shame in relation to their substance use problems. Some of the therapists’ descriptions indicate that in general terms, there is a qualitative difference when it comes to the relationship with internet-based patients compared to face-to face patients. It is harder to remember patients in internet-based treatment and easier to confuse them with other patients. Several of the therapists say that it is less tiring to work with internet-based treatment. Increased focus on the tasks and goals of the treatment could be one explanation, another might be that you do not get so emotionally involved. This is in line with therapists’ experiences in Bengtsson [23] who describe face-to-face therapy as a stronger experience than internet-based treatment, that the CBT treatment material gives more structure and focus compared to face-to-face therapy and that internet-based treatment provides more work-time control and might buffer therapist exhaustion. The therapists talk about the relative high degree of drop-outs from internet-based treatment. Drop-out rates have been found to be higher in internet-based interventions, compared to face-to-face interventions [33]. One explanation mentioned in the interviews is that patients in internet-based treatment are not so concerned about their therapists. If you feel you do not need more treatment or that treatment is not working, it seems to be much easier to drop out from internet-based programs than not showing up for face-to-face treatment. But is this a problem? As described earlier, this is one of the aspects of increased autonomy in treatment. Patients who continue in treatment due to obligations to the therapist makes the treatment purposeless. The therapists also say they “take up less space in” treatment while patients in previous research feel empowered. There seem to be a shift in responsibility but maybe also in power in internet-based treatment to a more equal relationship, similar to the partnership described in motivational interviewing [34]. The patient’s need of a relationship with the therapist seem to vary. Some patients work on their own with the written material and, according to the therapists, are not so reliant on feedback, response or communication from and with the therapist. Other patients communicate more and seem to want a higher degree of relation with the therapist. This is in line with previous studies that describe a continuum in internet-based treatment from close contact or high level of independence [12]. This could be an issue of different needs of different patients, or an issue of settings. Self-help interventions might be effective for motivated clients while more therapist-assisted treatments may be more suited for treatment at clinical level [5]. But rather than being distinct groups of patients, all patients might need both of the elements that Knowles [35] describes as connection and collaboration. Internet-based treatment might help therapists to find the best balance for each patient.

A close relationship between the therapist and the patient is, however, not the same as a good alliance. The therapist who is happy with the “distance” and happy with not having to think about patients after office hours in internet-based treatment might have a good alliance with a patient who is equally happy with an internet-based program where the main focus is your own work with the written material. Here, the reflections provided by the therapist might not be of that great importance to the patient. Alliance is built on mutual trust and acceptance, agreement about the goals, and agreement about the tasks for therapy [17] and according to previous research there is little in the internet-based form per se that prevents good alliances [20]. The process of writing and the perceived anonymity have been described by therapist as important factors for the patients to develop trust during online therapy [22]. Recurring in the interviews are the therapists descriptions on the importance of finding the correct “tune” with the patient, or a common “lingo” as described in an earlier quote. This can be interpreted in terms of the therapists’ strategies to accomplish a foundation for the bond that is important for alliance [17]. One challenge lies within the program-based treatment that most of the therapists in this study have worked with. Establishing an alliance is described as harder with patients who only work with the program-material and otherwise provide limited writing to the therapist. Communicating in between exercises is important for developing a personal touch that some of the therapists try to give. This could be seen as trying to establish trust or expressing acceptance. However, there are some examples in the interviews that exemplifies occasions when the mutual agreement is unclear. The therapists talk about patients who have been surprised when they have got a response, as if they have been surprised that there is an actual person on the other side of the screen. Another interpretation is that patients in internet-based treatment to a less extent experience themselves as “consumers of treatment” as one therapist puts it. Internet-based treatment is maybe seen as less demanding and that is perhaps a reason for why they have decided to try it in the first place. One therapist described a patient who had not even realized that the online program was treatment. This is, however, not the common narrative in the interviews. On the contrary, there seems to be an agreement on both goals and tasks for treatment, according to the therapists. This is probably affected by the setting and the CBT-programs that the therapists in this study have worked with. Agreement on the closeness or degree of alliance might be an important factor to consider in Internet-based treatment since it is less given than in face-to-face. Establishing a close therapeutic alliance might be more complex and maybe less expected by patients in internet-based treatments. This will probably change as the technology develops further and patient and therapists get more and more comfortable in using different forms of internet-based communication.

### Implications for future research and practice

Different aspects of the alliance seem to be important in internet-based treatment compared to face-to-face. In a study that measured alliance, different facets were important for treatment success in internet-based treatment compared to group-treatment [36]. Future studies that measure alliance in internet-based treatment should observe such aspects. Future internet-based treatments might try to use systems for feedback from patients [37] that can help therapists prevent drop-out that are related to therapist-client alliance. Current definitions of therapeutic alliance seem adapted to face-to-face meetings and future definitions could try to incorporate the experience of internet-based therapy. Furthermore, we need to broaden our insights on patient’s perspectives on internet-based treatment of substance use and on how they perceive aspects as, e.g. alliance, in order to better understand the of internet-based treatment.

## Limitations

The findings in this study are limited by the sample of therapists, which was based on availability. Psychologists working with alcohol use were overrepresented. They all lived a big European city, Stockholm, and worked with CBT-based programs. This is one of relatively few qualitative studies of therapist perceptions of internet-based treatment, and the first in the field of substance use. A strength compared to other similar studies is that both therapists that primarily work on the internet, as well as therapists that no longer do it, participated in the group interviews. Another possible limitation with this study is how data was collected. In focus group interviews, there is a risk of peer pressure within the group to provide similar answers, and dominant participants can take over the interview, During the interviews, participants were encouraged to engage in the conversation and directed follow up questions were used to involve the more silent participants.

Furthermore, our own understandings and preconceptions concerning alcohol treatment, CBT-programs and internet-based treatment might be another limitation. One of us (author 2, MJ) has been involved in clinical work with treatment for problematic alcohol use both face-to-face and online. He is also part of the research team responsible for evaluating some of the programs the therapists in our study have worked with. Therefore, we decided that only author 1 (VE) would participate in the group interviews. We have tried to strengthen the reliability of our study by combining our different positions, i.e. the position of being very familiar or inside and the more distanced outsider position.

## Conclusion

In this article, we have investigated internet-based treatment for substance use from the therapists’ perspectives. We have tried to understand differences between traditional treatment and internet-based treatment and what these differences mean for alliance in internet-based treatment. Especially when working with CBT-programs, there may be a lot of similarities between traditional face-to-face treatment and internet-based treatment. However, as our study shows, there are also important differences. The written form creates something qualitatively different from regular face-to-face meetings between patients and therapists. The written form changes what time is in treatment, how it can be used and how it affects therapists’ presence. From the therapists’ perspective, special considerations and skills are needed to be a good therapist on-line. How alliance is achieved and maintained in internet-based treatment seem to be different than in face-to-face treatment. Agreement with the patient on the closeness of the relationship might be an important factor to consider in internet-based treatment.

## Supplementary information

**Additional file 1.** Interview guide.

**Additional file 2.** COREQ checklist.

## Data Availability

The qualitative data analyzed during the current study have not been made publicly available, in order to protect the participants identities. The transcribed focus group interviews are in Swedish. Translated pseudonymized data are available from the corresponding author on reasonable request.
