# Supplementary material for: Sort of a nice distance: a qualitative study of the experiences of therapists working with internet-based treatment of problematic substance use
Source: Addict Sci Clin Pract. 2019 Nov 27;14:44. doi: 10.1186/s13722-019-0173-1 (PMC6880358; doi:10.1186/s13722-019-0173-1)
Supplement: Supplementary file 1 — Additional file 1. Interview guide. [file 13722_2019_173_MOESM1_ESM.docx]

Interview guide

1. Who are you and what are your experiences from working with treatment for problematic use of alcohol/cannabis, etc.?
2. Did you get any special training before you started working with internet-based treatment?
3. What do you think is different with internet-based treatment?
4. What is worse/better?
5. How do technology affect your work?
6. Do you reach other patients with internet-based treatment than in face-to-face treatment?
7. Do patients need to be different/have different skills to be suitable for internet-based treatment?
8. Do you as a therapist need to do something different or be different?
9. Do you want to add anything?
